# Association between triglyceride glucose-body mass index and cardiovascular outcomes in patients undergoing percutaneous coronary intervention: a retrospective study

**DOI:** 10.1186/s12933-023-01794-8

**Published:** 2023-03-30

**Authors:** Yang Cheng, Zhen Fang, Xinxin Zhang, Yuchen Wen, Jiaqi Lu, Shenghu He, Bing Xu

**Affiliations:** 1 Clinical Medical College, Yangzhou University, Yangzhou, 225001 China; 2 Present Address: The Yangzhou School of Clinical Medicine, Dalian Medical University, Dalian, 116044 China; 3 Present Address: Department of Cardiology, Northern Jiangsu People’s Hospital, Yangzhou University, Yangzhou, 225001 China

**Keywords:** Triglyceride glucose-body mass index, Insulin resistance, Major adverse cardiac and cerebrovascular events, Coronary artery disease, Percutaneous coronary intervention, Drug-eluting stent

## Abstract

**Background:**

The triglyceride glucose-body mass index (TyG-BMI index) has been considered a reliable surrogate measure of insulin resistance; however, its ability to predict the incidence of cardiovascular disease in individuals with coronary artery disease (CAD) remains uncertain. The aim of this study was to demonstrate the correlation between the TyG-BMI index and cardiovascular incidence.

**Methods:**

A total of 2533 consecutive participants who underwent percutaneous coronary intervention (PCI) and drug-eluting stent (DES) implantation were included. Data from 1438 patients was analyzed in the study. The endpoint was defined as a composite of acute myocardial infarction, repeat revascularization, stroke, and all-cause mortality (major adverse cardiac and cerebrovascular events, MACCEs) at 34-month follow-up. The formula for calculating the TyG-BMI index is ln [fasting triglyceride (mg/dL) × fasting blood glucose (mg/dL)/2] × BMI.

**Results:**

Among the 1438 participants, 195 incident patient cases of MACCEs were ascertained. The incidence of MACCEs showed no statistically significant differences in the TyG-BMI index tertiles in the overall population. Further exploratory subgroup analysis and multivariable logistic regression analysis revealed a linear relationship between the TyG-BMI index (per 1 SD increased) and MACCEs in the elderly patients (OR = 1.22, 95% CI 1.011–1.467, p = 0.038) and in the female patients (OR = 1.33, 95% CI 1.004–1.764, p = 0.047). The addition of the TyG-BMI index to traditional risk factor models in elderly and female patients did not improve risk prediction for MACCEs.

**Conclusion:**

A higher TyG-BMI index was proportionally related to an increased incidence of MACCEs in the elderly or female patients. However, the inclusion of the TyG-BMI index did not provide better predictive performance for MACCEs in the elderly, specifically in female patients.

**Supplementary Information:**

The online version contains supplementary material available at 10.1186/s12933-023-01794-8.

## Background

Coronary artery disease (CAD) has been considered the primary cause of death and is continuously raising the expense of health care and the burden on societal productivity not only in China but also all around the world [1, 2]. Although superior optimized strategies for revascularization and medication treatment have been developed and applied recently, there is still a high incidence of recurrent cardiovascular events [3, 4]. Therefore, it is of great clinical importance to improve the risk stratification to facilitate the management of patients with CAD, which is also a great challenge [5].

Insulin resistance (IR) means the diminished or impaired sensitivity to endogenous and exogenous insulin in insulin-dependent organs and tissues [6]. IR is also a critical hazard factor for the progress of type 2 diabetes mellitus (T2DM) and cardiovascular diseases [7, 8]. Multiple methods for IR assessment have been developed. However, the clinical application is limited [7, 9, 10]. Besides, the triglyceride glucose index (TyG index), which is calculated by considering both fasting triglycerides and fasting blood glucose level, has been shown as a symbol highly correlated to hyper-insulinemic euglycemic clamp (HEC) as well as the homeostatic model assessment of insulin resistance index (HOMA-IR) [11, 12]. Studies have revealed that the TyG index is related to pathophysiological processes including arterial stiffness and coronary artery calcification, which are associated with cardiovascular disorders like CAD and hypertension [13–20].

Obesity, identified by body mass index (BMI), is another principal factor linked to IR [21]. A combination of TyG and BMI (TyG-BMI index) was revealed to be in good accordance with HOMA-IR for IR assessment in the Korean population [6] and Chinese population [22]. Recently, one study showed that the TyG-BMI index was linearly linked with ischemic stroke [23]. These studies indicate that the TyG-BMI index may have a potential association with cardiovascular events in patients with CAD. However, very few studies focused on the influence of the TyG-BMI index on cardiovascular outcomes in those who underwent DES implantation. Consequently, current work aims to explore the impact of the baseline TyG-BMI index on cardiovascular outcomes in individuals with CAD undergoing PCI and to evaluate whether combining the TyG-BMI index with traditional risk indicators improves risk stratification.

## Methods

### Study population

**Fig. 1:**
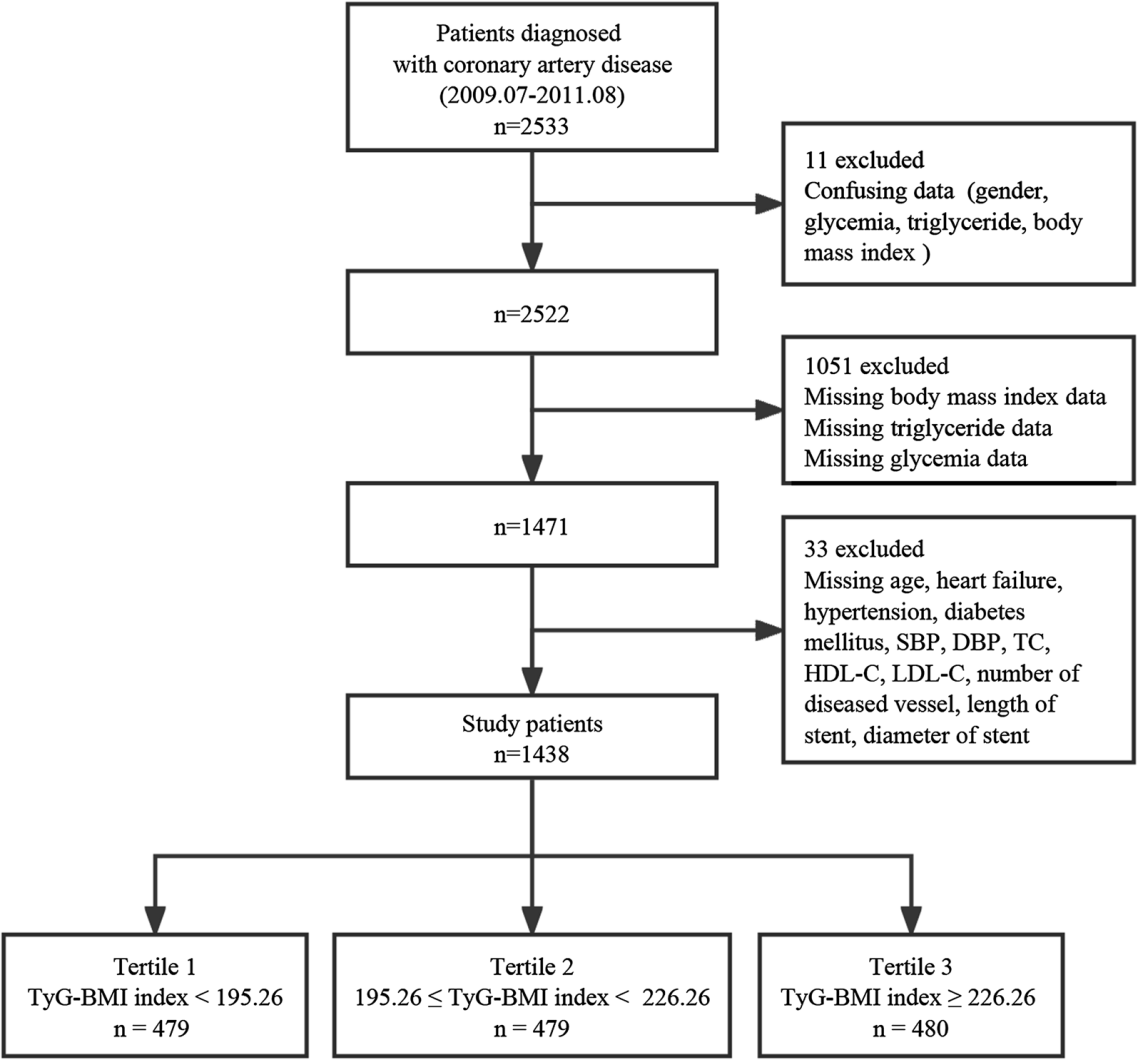
Flowchart of participant selection. DBP diastolic blood pressure; HDL-C, high-density lipoprotein cholesterol; LDL-C, low-density lipoprotein cholesterol; SBP, systolic blood pressure; TC, total cholesterol; TG, triglyceride; TyG-BMI index, triglyceride glucose-body mass index

Yao HM et al. provided the data used in the research [24], which is accessible through Dryad (https://doi.org/10.5061/dryad.13d31) [25]. The Ethics Committee at Zhengzhou University's First Affiliated Hospital authorized this research in line with the Declaration of Helsinki, and a waiver of informed consent was granted. Since research ethics has already approved the public policy statement underlying the dataset, no ethical declaration was necessary for the current study. The original study was designed to assess the prognosis of patients with coronary artery disease. All study participants who underwent PCI and were treated with DES between July 2009 and August 2011 (n = 2533) were included, and all completed a median of 29.8 months (quartiles, 25.6–34 months) of follow-up. Coronary angiography and PCI were conducted according to standard procedures and guidelines. All patients were administered loading doses of aspirin (300 mg) and clopidogrel (300 mg) before the coronary intervention, except those who had already been treated with antiplatelet medication. The surgeon decided the selection of procedure strategy, stenting techniques, stent type, and use of glycoprotein IIb/IIIa receptor inhibitors or intravascular ultrasound. All patients were treated with standard dual antiplatelet therapy (aspirin 100 mg/day and clopidogrel 75 mg/day) after PCI for at least one year, constantly [24]. After excluding confusing or missing data, data covering 1438 patients were used for the current analyses (Fig. 1).

### Data collection and definitions

Demographic data (age, gender, body mass index), comorbid diseases (heart failure, atrial fibrillation, previous acute myocardial infarction, previous stroke, PCI, hypertension, diabetes mellitus, smoking), and diagnostic information were obtained at admission. Routine laboratory tests, including glycemia, creatinine, uric acid, bilirubin, low-density lipoprotein cholesterol (LDL-C), high-density lipoprotein cholesterol (HDL-C), total cholesterol (TC), and triglyceride (TG), were performed using fasting blood samples. The laboratory data were gathered from medical records. The angiographic data, including the operative approach, number of diseased vessels, location of target lesions (left main coronary artery (LM), left anterior descending (LAD), left circumflex artery (LCX), and right coronary artery (RCA)), characteristics of lesions (occlusion, chronic total occlusions (CTO), ostial lesion, and bifurcation lesion), number of stents, length and diameter of stents, and number of treated vessels, were also collected. Besides that, information about medications (aspirin, clopidogrel, beta blockers, angiotensin-converting enzyme inhibitors (ACEI), calcium channel blockers (CCB), and statins) was obtained. All data were collected retrospectively using a standardized data collection form. The follow-up information was gathered through the outpatient clinic, readmission, or phone contact.

The TyG-BMI index was ln [TG (mg/dL) × FBG (mg/dL)/2] × BMI [22]. The elderly patients were those aged over sixty years [26]. Smokers were defined as those who had smoked in the last decade. The history of diabetes mellitus was defined as treatment with diabetes medication or self-reported diabetes mellitus. The history of hypertension was defined as treatment with antihypertensive drugs or a self-reported hypertension. Repeat revascularization was revascularization with PCI or bypass surgery during the follow-up.

### Endpoints

The endpoint of this study was major adverse cardiac and cerebrovascular events (MACCEs) at 34-month follow-up. The MACCEs included acute myocardial infarction, repeat revascularization, all-cause mortality, and stroke.

### Statistical analysis

All participants were separated into tertiles according to the TyG-BMI index levels: tertile 1 (n = 479, TyG-BMI index < 195.26), tertile 2 (n = 479, 195.26 ≤ TyG-BMI index < 226.26), and tertile 3 (n = 480, TyG-BMI index ≥ 226.26), and the characteristics were depicted. Categorical variables were shown as numbers (n) and percentages (%) and were assessed using chi-square tests. Continuous variables were presented as the mean ± standard deviation for a normal distribution or the median (interquartile range) for a non-normal distribution. They were analyzed using one-way ANOVA and the Kruskal–Wallis test, respectively.

We standardized (Z-score) the TyG-BMI index, then included it in the univariate and multivariate logistic analyses to investigate the impact of an increase in the TyG-BMI per SD on MACCEs. Before constructing the multivariate model, we examined the collinearity between the TyG-BMI index and other variables by calculating the generalized variance inflation factor (GVIF). The variable was considered significant for collinearity if the GVIF^(1/2Df) was equal to or greater than 2 (Df, degrees of freedom). In the study, we selected the covariates based on the following principles: (1) if the variable was introduced into the basic model or was removed from the full model, the matched odds ratio would change more than 10%; (2) variables with a p-value < 0.1 in the univariate model; and (3) variables were considered based on previous findings, clinical significance, and the number of outcome events. Following the criteria above, we adjusted for age, heart failure, history of acute myocardial infarction (AMI), hypertension, diabetes, left ventricular ejection fraction (LVEF), ACEI, LM, CTO, number of stents, and diameter of stents in the multivariate logistic analyses for the overall population.

Additionally, subgroup analyses were done to explore if the association was modified by gender, age, hypertension, and diabetes mellitus. Interactions between the TyG-BMI index and the variables above were examined separately. Then we performed univariate and multivariate logistic analyses to describe the relationship between TyG-BMI per SD and MACCEs in the elderly patients and the female patients separately. According to the rules for covariate screening above, we adjusted for age, heart failure, history of AMI, ACEI, and uric acid (per 20 units) for elderly patients and controlled for age, history of AMI, diabetes mellitus, CTO, uric acid (per 20 units), and number of treated vessels for female patients in the multivariate model. Restricted cubic splines were applied to evaluate potential nonlinear associations between the TyG-BMI index and MACCEs in the gender and age subgroups. Ultimately, the receiver operating characteristic (ROC) curves were generated to assess whether adding the TyG-BMI index improves the predictive ability of the model containing traditional risk indicators. The area under the curve (AUC) comparisons between the models were evaluated with DeLong’s test. Furthermore, two other measures, net reclassification improvement (NRI) and integrated discrimination improvement (IDI), were computed to assess the additional predictive information of the TyG-BMI index.

Findings are reported by odds ratios (OR) and 95% confidence intervals (CI). All tests were two-tailed, and statistical significance was defined as P < 0.05. All analyses were conducted using the R Statistical Software (http://www.R-project.org, The R Foundation), Free Statistics software versions 1.7, and MedCalc version 20.0.22.

## Results

### Characteristics of the included and excluded patients

After excluding the confusing data, 1084 patients were excluded due to the missing data. Considering the high proportion of missing values, the characteristics of the included patients and the excluded patients were compared (Additional file 1: Table S1). There were no significant differences in the age and gender distributions between the two groups. The analyzed patients had a higher proportion of diabetes, smoking, and non-ST elevation acute coronary syndromes (NSTE-ACS); in contrast, the excluded patients had a higher percentage of previous PCI or CABG and stroke. No significant differences were observed in the angiographic characteristics except for the higher proportion of multi-vessel lesions. We also noticed that more patients in the excluded group received follow-up angiography, and cardiac death and repeat revascularization occurred more frequently in the group.

### Characteristics of the population by TyG-BMI index

A total of 1438 patients were finally included, with a mean age of 60.1 ± 11.1 years, a gender ratio (male/female) of 2.18, and an average TyG-BMI index of 213.7. The characteristics of the patients by tertile of the TyG-BMI index are shown in Table 1. The percentage of females, those with a history of hypertension and diabetes, and those treated with ACEI or CCB increased in parallel with the TyG-BMI index. Patients with higher levels of the TyG-BMI index tended to have higher levels of BMI, TG, TC, and LDL-C and were more likely to have a lower proportion of smoking and a lower level of creatinine and HDL-C. The higher TyG-BMI index group presented with a larger proportion of NSTE-ACS, a higher frequency of multi-vessel lesions, RCA lesions, and more stent implantation; besides, the length of the stents was longer. However, no discernible variations were displayed in age, bilirubin, uric acid, medical history of heart failure, atrial fibrillation, AMI, stroke, or PCI.

**Table 1:** Baseline characteristics of the study population by tertiles of TyG-BMI index

	Tertile 1 (n = 479)	Tertile 2 (n = 479)	Tertile 3 (n = 480)	P value
Demographics				
Gender, female, (%)	65 (13.6)	167 (34.9)	220 (45.8)	** < 0.001**
Age, years	59.4 ± 11.4	60.5 ± 11.1	60.3 ± 10.7	0.254
BMI, (Kg/m^2^)	20.2 ± 2.1	23.7 ± 1.7	27.6 ± 2.9	** < 0.001**
Medical history				
Heart failure, (%)	51 (10.6)	46 (9.6)	61 (12.7)	0.294
Atrial fibrillation, (%)	13 (2.7)	8 (1.7)	4 (0.8)	0.083
Previous AMI, (%)	49 (10.2)	39 (8.1)	41 (8.5)	0.487
Previous stroke, (%)	24 (5)	21 (4.4)	18 (3.8)	0.635
Previous PCI, (%)	32 (6.7)	24 (5)	24 (5)	0.429
Hypertension, (%)	175 (36.5)	235 (49.1)	309 (64.4)	** < 0.001**
Diabetes mellitus, (%)	66 (13.8)	105 (21.9)	157 (32.7)	** < 0.001**
Smoking, (%)	212 (44.3)	143 (29.9)	138 (28.7)	** < 0.001**
Clinical presentation				**0.001**
SA, (%)	58 (12.1)	56 (11.7)	59 (12.3)	
NSTE-ACS, (%)	265 (55.3)	313 (65.3)	316 (65.8)	
STEMI, (%)	156 (32.6)	110 (23)	105 (21.9)	
LVEF, (%)	60.3 ± 7.4	61.0 ± 7.4	62.0 ± 6.9	**0.011**
Laboratory data				
Glycemia, mmol/L (IQR)	5.0 (4.6, 5.7)	5.2 (4.7, 6.3)	5.8 (5.0, 7.6)	** < 0.001**
Creatinine, umol/L (IQR)	71.0 (61.0, 85.0)	69.0 (57.0, 81.0)	66.0 (55.0, 80.0)	** < 0.001**
Uric acid, umol/L (IQR)	302.0 (246.8, 355.0)	291.0 (244.5, 354.0)	298.0 (240.2, 356.0)	0.669
Bilirubin, umol/L (IQR)	8.3 (5.9, 11.6)	8.6 (5.8, 12.2)	8.8 (5.9, 11.7)	0.791
TG, mmol/L (IQR)	4.0 (3.4, 4.7)	4.1 (3.5, 4.8)	4.5 (3.8, 5.2)	** < 0.001**
TC, mmol/L (IQR)	1.3 (1.0, 1.8)	1.5 (1.2, 2.1)	2.2 (1.5, 3.0)	** < 0.001**
HDL-C, mmol/L (IQR)	1.1 (0.9, 1.3)	1.0 (0.9, 1.2)	1.0 (0.8, 1.2)	** < 0.001**
LDL-C, mmol/L (IQR)	2.6 (2.0, 3.2)	2.6 (2.0, 3.3)	2.8 (2.2, 3.5)	** < 0.001**
Treatment				
Aspirin, (%)	476 (99.4)	470 (98.3)	470 (98.1)	0.207
Clopidogrel, (%)	460 (96)	460 (96)	470 (97.9)	0.172
Beta blocker, (%)	325 (67.8)	348 (72.7)	329 (68.5)	0.217
ACEI, (%)	247 (51.6)	282 (58.9)	309 (64.5)	** < 0.001**
CCB, (%)	79 (16.5)	121 (25.3)	152 (31.7)	** < 0.001**
Statin, (%)	450 (93.9)	452 (94.4)	441 (91.9)	0.251
Radial artery access, (%)	468 (97.7)	467 (97.5)	471 (98.1)	0.797
Number of diseased vessels, (%)				0.098
1-vessel disease	200 (41.8)	168 (35.1)	163 (34)	
2-vessel disease	173 (36.1)	184 (38.4)	190 (39.6)	
3-vessel disease	106 (22.1)	127 (26.5)	127 (26.5)	
Location of target lesions				
LM, (%)	19 (4)	17 (3.5)	10 (2.1)	0.22
LAD, (%)	396 (82.7)	414 (86.4)	393 (81.9)	0.126
LCX, (%)	232 (48.4)	233 (48.6)	245 (51)	0.668
RCA, (%)	217 (45.3)	250 (52.2)	260 (54.2)	**0.016**
Characteristics of lesions				
Occlusion, (%)	69 (14.4)	62 (12.9)	64 (13.3)	0.791
CTO, (%)	41 (8.6)	48 (10)	31 (6.5)	0.134
Ostial lesion, (%)	72 (15)	48 (10)	50 (10.4)	**0.028**
Bifurcation lesion, (%)	99 (20.7)	72 (15)	78 (16.2)	0.053
Number of treated vessels, (%)				**0.028**
1	303 (63.3)	269 (56.2)	261 (54.4)	
2	133 (27.8)	166 (34.7)	179 (37.3)	
≥ 3	43 (9)	44 (9.2)	40 (8.3)	
Number of stents, (%)				**0.042**
1	209 (43.6)	188 (39.2)	168 (35)	
2	146 (30.5)	143 (29.9)	149 (31)	
≥ 3	124 (25.9)	148 (30.9)	163 (34)	
Length of stents, (mm)	46.1 ± 29.7	49.3 ± 33.2	51.1 ± 30.9	**0.041**
Diameter of stents, (mm)	3.2 ± 1.3	3.1 ± 1.3	3.1 ± 0.4	0.387

Data are shown as mean ± standard deviation (SD) or median (IQR) for continuous variables and proportions (%) for categorical variables

ACEI, angiotensin converting enzyme inhibitor; AMI, acute myocardial infarction; AVB, atrioventricular block; BMI, body mass index; CCB, calcium channel blocker; CABG, coronary artery bypass graft; COPD, chronic obstructive pulmonary disease; CTO, chronic total occlusions; HDL-C, high density lipoprotein cholesterol; LAD, left anterior descending; LDL-C, low density lipoprotein cholesterol; LM, left main coronary artery; LCX, left circumflex artery; LVEF, left ventricular ejection fraction; NSTE-ACS, non-ST elevation acute coronary syndromes; PCI, percutaneous coronary intervention; RCA, right coronary artery; SA, stable angina; STEMI, ST-segment myocardial infarction; TC, total cholesterol; TG, triglyceride; TyG-BMI, triglyceride glucose-body mass index

P values in bold are < 0.05

### TyG-BMI index and clinical outcomes in the overall population

The occurrence of MACCEs and individual events are summarized in Table 2. A total of 195 patients (13.6%) had at least one MACCE during follow-up, including 60 (12.5%) patients in the Tertile 1 group, 64 (13.4%) in the Tertile 2 group, and 71 (14.8%) in the Tertile 3 group. The incidence of MACCEs and the individual events showed no significant difference among the three groups. Univariate and multivariate logistic regression analyses were performed to determine the predictors for MACCEs in the overall population (Table 3). The rules of covariate screening were described in detail in the “Methods” section, and we presented the information for selection in Additional file 1: Table S2. The collinearity was assessed before the analyses (Additional file 1: Table S2). We excluded BMI, TC, TG, LDL-C, LCX, RCA, and length of the stents from analyses due to high collinearity with the TyG-BMI index. The risk factors for MACCEs were age, LVEF, the use of ACEI, CTO, LM, stent numbers, and stent diameters (all p < 0.05). However, no statistically significant connection between the TyG-BMI index (per 1 SD increase) and MACCEs was found either in univariate analysis (OR = 1.12, 95% CI 0.97–1.3, p = 0.134) or in multivariate analysis (OR = 1.11, 95% CI 0.91–1.37, p = 0.304). Additionally, we performed subgroup analyses to investigate the impacts of various potential confounding factors, including gender, age, hypertension, and diabetes mellitus (Table 4). We found significant interactions between the subgroups stratified by age and the effect of the TyG-BMI index on the MACCEs (p-value for interaction = 0.019). A similar result was also found in the female subgroup, though there was no statistical significance (p-value for interaction = 0.068).

**Table 2:** Incidence of clinical outcomes in the overall population during follow-up

	Tertile 1 (n = 479)	Tertile 2 (n = 479)	Tertile 3 (n = 480)	P value
MACCEs, (%)	60 (12.5)	64 (13.4)	71 (14.8)	0.584
All cause death, (%)	26 (5.4)	34 (7.1)	30 (6.2)	0.566
Cardiac death, (%)	4 (0.8)	1 (0.2)	1 (0.2)	0.351
AMI, (%)	19 (4)	19 (4)	20 (4.2)	0.984
Revascularization, (%)	25 (5.2)	26 (5.4)	28 (5.8)	0.914
Stroke, (%)	3 (0.6)	6 (1.3)	12 (2.5)	0.048

Data are shown as proportions (%)

MACCEs was defined as a composite of acute myocardial infarction, revascularization, stroke, and all-cause mortality

MACCEs, major adverse cardiac and cerebrovascular events; AMI, acute myocardial infarction

**Table 3:** Univariate and multivariate analysis for predictors of MACCEs in overall population

	Univariate analysis		Multivariate analysis	
	OR (95% CI)	P value	OR (95% CI)	P value
TyG-BMI (per 1 SD)	1.12 (0.97–1.3)	0.134	1.11 (0.91–1.37)	0.304
Age	1.05 (1.03–1.06)	** < 0.001**	1.04 (1.02–1.06)	** < 0.001**
Heart failure	1.74 (1.14–2.65)	**0.01**	1.29 (0.76–2.2)	0.339
Previous AMI	1.8 (1.14–2.83)	**0.011**	1.2 (0.67–2.12)	0.54
Hypertension	1.35 (0.99–1.83)	0.055	0.93 (0.62–1.41)	0.744
Diabetes mellitus	1.71 (1.23–2.38)	**0.001**	1.46 (0.95–2.24)	0.088
LVEF	0.95 (0.93–0.98)	** < 0.001**	0.97 (0.94–0.99)	**0.007**
ACEI	2.04 (1.46–2.85)	** < 0.001**	2.03 (1.31–3.14)	**0.001**
LM	1.81 (0.88–3.71)	0.104	3.15 (1.3–7.62)	**0.011**
CTO	2.84 (1.85–4.37)	** < 0.001**	1.97 (1.08–3.62)	**0.028**
Number of stents				
1	1(ref)		1(ref)	
2	0.77 (0.51–1.15)	0.202	0.52 (0.32–0.86)	**0.012**
≥ 3	1.64 (1.16–2.32)	**0.005**	0.97 (0.61–1.52)	0.878
Diameter of stents	0.58 (0.4–0.85)	**0.004**	0.55 (0.33–0.91)	**0.021**

OR, odds ratio; CI, confidence interval; SD, standard deviation

Abbreviations as in Table 1

P values in bold are < 0.05

**Table 4:** Subgroup analysis for association between TyG-BMI index (per 1 SD) and MACCEs

Subgroup	n total	n event (%)	Unadjusted OR (95% CI)	Unadjusted P value	Adjusted OR (95% CI)	Adjusted P value	P for interaction
Gender							**0.068**
Female	452	64 (14.2)	1.39 (1.08–1.78)	**0.01**	1.30 (0.97–1.73)	**0.076**	
Male	986	131 (13.3)	0.98 (0.8–1.2)	0.846	0.93 (0.74–1.16)	0.524	
Age, years							**0.019**
Age < 60	647	58 (9)	0.87 (0.65–1.15)	0.333	0.76 (0.55–1.04)	0.089	
Age ≥ 60	791	137 (17.3)	1.21 (1.01–1.44)	**0.034**	1.22 (1–1.48)	**0.051**	
Hypertension							0.173
No	719	85 (11.8)	0.97 (0.76–1.24)	0.805	0.94 (0.72–1.24)	0.680	
Yes	719	110 (15.3)	1.16 (0.96–1.41)	0.130	1.13 (0.92–1.39)	0.246	
Diabetes mellitus							0.331
No	1110	133 (12)	1.02 (0.85–1.23)	0.836	1.01 (0.83–1.24)	0.898	
Yes	328	62 (18.9)	1.18 (0.91–1.54)	0.217	1.15 (0.87–1.53)	0.337	

Unadjusted model: no covariates were adjusted; Adjusted model: adjusted for age, heart failure, previous AMI, hypertension, diabetes mellitus, ACEI, CTO, diameter of stents, number of diseased vessels

### The linear relationship between the TyG-BMI index and MACCEs in the elderly population

**Fig. 2:**
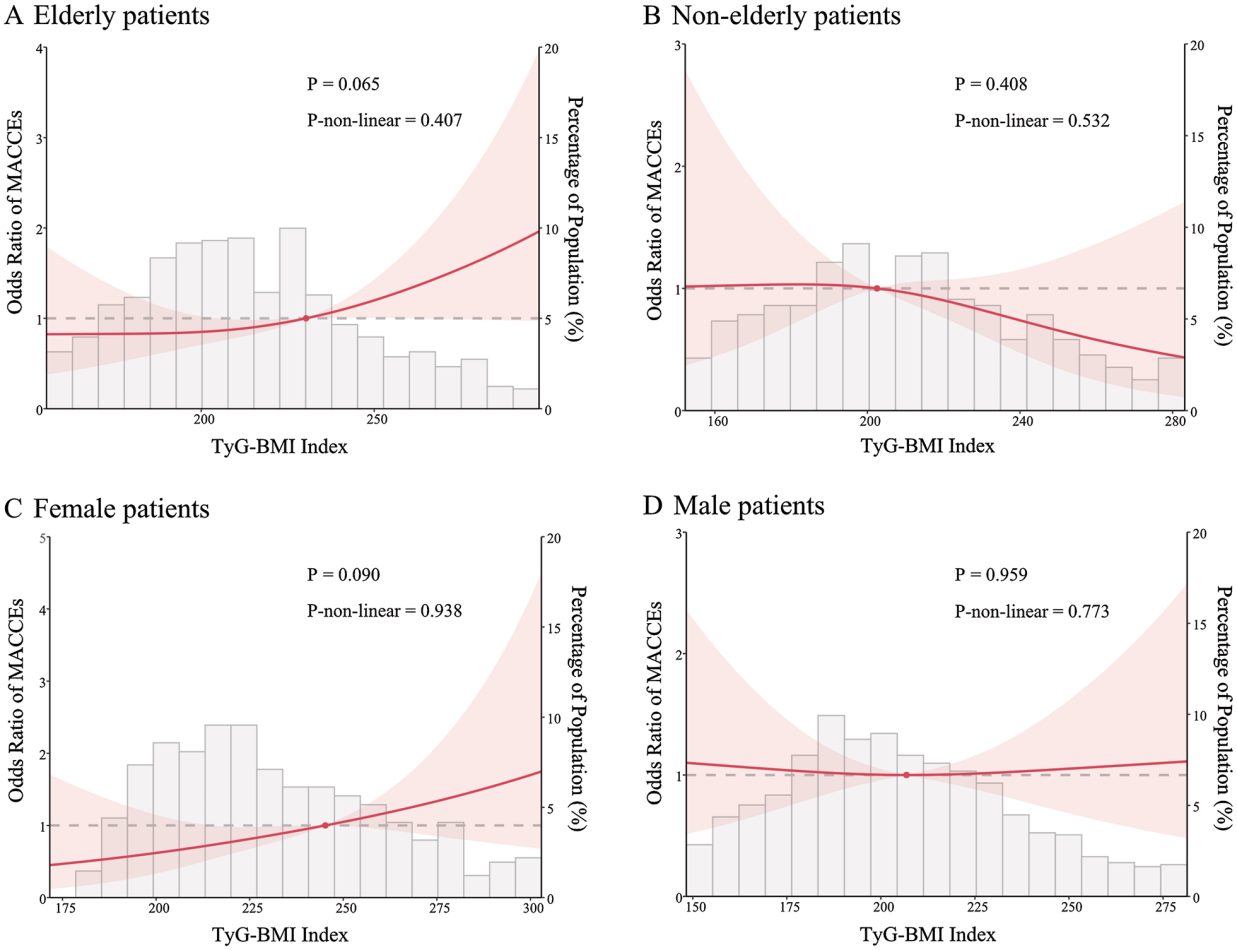
The relationship between the TyG-BMI index and MACCEs. **A** Relationship in the elderly patients. Adjusted for all covariates in Table 4. **B** Relationship in the nonelderly patients. **C** Relationship in the female patients. Adjusted for all covariates in Table 5. **D** Relationship in the male patients. Only 95% of the data is displayed. Odds ratios are indicated by solid lines and 95% CIs by shaded areas

Based on the findings in the subgroup analyses, we further analyzed the association between the TyG-BMI index and MACCEs in elderly patients. Similarly, we conducted collinearity analysis, variable screening (Additional file 1: Table S3), and logistic analyses. We found that the TyG-BMI index (per 1 SD increase) was correlated with a higher risk of MACCEs in the patients aged over 60 years old (OR = 1.21, 95% CI 1.02–1.44, p = 0.034), and the association remained significant (OR = 1.22, 95% CI 1.01–1.47, p = 0.038) even after controlling for the possible confounding variables (Table 5). A restricted cubic spline model was established to define the nonlinear relationship between the TyG-BMI index (per 1 unit) and MACCEs in elderly individuals. The model showed a linear association (p for non-linearity = 0.407) (Fig. 2A). There was no significant association between the TyG-BMI index and MACCEs in the nonelderly subgroup, though the restricted cubic splines model showed a linear relationship (p for non-linearity = 0.532) (Fig. 2B). In addition, we compared the individual outcomes between the lower TyG-BMI index group (< 210.76) and the higher TyG-BMI index group (≥ 210.76), however, no significance was found in all-cause death, cardiac death, AMI, repeat revascularization, or stroke (Table 6).

**Table 5:** Univariate and multivariate analysis for predictors of MACCEs in elderly patients

	Univariate analysis		Multivariate analysis	
	OR (95% CI)	P value	OR (95% CI)	P value
TyG-BMI (per 1 SD)	1.21 (1.02–1.44)	0.034	1.22 (1.01–1.47)	0.038
Age	1.08 (1.05–1.11)	< 0.001	1.07 (1.04–1.10)	< 0.001
Heart failure	1.98 (1.219–3.229)	0.006	1.81 (1.09–3.01)	0.023
Previous AMI	2.15 (1.27–3.64)	0.004	1.98 (1.14–3.44)	0.015
ACEI	2.38 (1.59–3.58)	< 0.001	1.94 (1.27–2.96)	0.002
Uric acid (per 20 units)	1.04 (1.01–1.08)	0.018	1.04 (1.00–1.07)	0.039

OR, odds ratio; CI, confidence interval; SD, standard deviation; Other abbreviations as in Table 1

**Table 6:** Incidence of clinical outcomes in elderly patients

Clinical outcomes	TyG-BMI index (< 210.76; n = 395)	TyG-BMI index (≥ 210.76; n = 396)	P value
All cause death	33 (8.4)	44 (11.1)	0.191
Cardiac death	2 (0.5)	1 (0.3)	0.624
Acute myocardial infarction	14 (3.5)	17 (4.3)	0.587
Revascularization	18 (4.6)	27 (6.8)	0.17
Stroke	3 (0.8)	10 (2.5)	0.051

Data are shown as proportions (%)

### The linear relationship between the TyG-BMI index and MACCEs in the female population

In the subgroup analyses, we observed the relationship between the TyG-BMI index (per 1 SD) and MACCEs in the female population. Then we constructed a logistic regression model to investigate the association further. The collinearity analysis and variable screening were shown in Additional file 1: Table S4. We included age, history of AMI, diabetes mellitus, CTO, uric acid (per 20 units), and number of treated vessels in the final model (Table 7). The results indicated that the baseline TyG-BMI index (per 1 SD) was significantly associated with MACCEs in the female population either in the univariate analyses (OR = 1.39, 95% CI 1.08–1.78, p = 0.010) or in the multivariate analyses (OR = 1.33, 95% CI 1.00–1.76, p = 0.047). Likewise, we fitted the restricted cubic spline model to assess the nonlinear relationship between the TyG-BMI index (per 1 unit) and MACCEs in female individuals. The model revealed a linear relationship (p for non-linearity = 0.938) (Fig. 2C). In the male patients, no association was observed between the TyG-BMI index and MACCEs (Fig. 2D). Furthermore, the incidence of single clinical outcomes was similar in the lower TyG-BMI index group (< 225.79) and the higher TyG-BMI index group (≥ 225.79) (Table 8).

**Table 7:** Univariate and multivariate analysis for predictors of MACCEs in female patients

	Univariate analysis		Multivariate analysis	
	OR (95% CI)	P value	OR (95% CI)	P value
TyG-BMI (per 1 SD)	1.39 (1.08–1.78)	0.010	1.33 (1.00–1.76)	0.047
Age	1.09 (1.05–1.13)	< 0.001	1.08 (1.04–1.12)	0.000
Previous AMI	2.52 (1.01–6.31)	0.048	2.31 (0.80–6.69)	0.124
Diabetes mellitus	2.43 (1.41–4.20)	0.001	2.34 (1.24–4.41)	0.008
CTO	2.89 (1.35–6.19)	0.006	2.336 (0.98–5.60)	0.057
Uric acid (per 20 units)	1.08 (1.03–1.14)	0.003	1.06 (1.01–1.11)	0.014
Number of treated vessels				
1	1 (ref)		1 (ref)	
2	0.63 (0.34–1.17)	0.143	0.47 (0.23–0.94)	0.033
≥ 3	1.95 (0.88–4.33)	0.100	1.80 (0.75–4.31)	0.191

OR, odds ratio; CI, confidence interval; SD, standard deviation; Other abbreviations as in Table 1

**Table 8:** Incidence of clinical outcomes in female patients

Clinical outcomes	TyG-BMI index (< 225.79; n = 395)	TyG-BMI index (≥ 225.79; n = 226)	P value
All cause death	20 (8.8)	17 (7.5)	0.607
Cardiac death	0 (0)	1 (0.4)	1
Acute myocardial infarction	5 (2.2)	10 (4.4)	0.189
Revascularization	8 (3.5)	11 (4.9)	0.482
Stroke	1 (0.4)	6 (2.7)	0.122

Data are shown as proportions (%)

### Incremental effect of the TyG-BMI index for predicting MACCEs

**Fig. 3:**
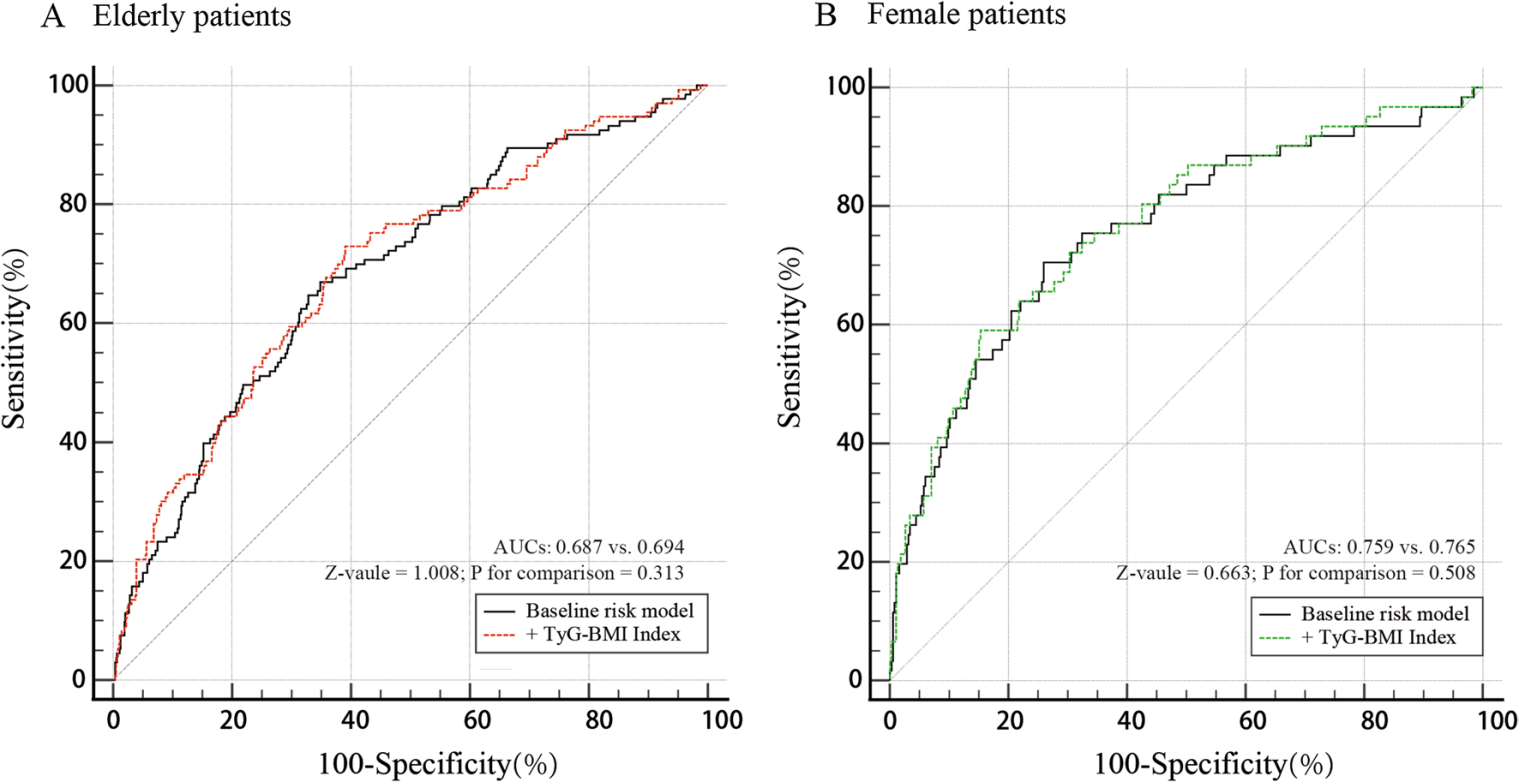
The receiver operating characteristic (ROC) curves of the TyG-BMI index as a marker to predict MACCEs. **A** Baseline risk model vs. + TyG-BMI index in the elderly patients. Baseline risk model includes age, heart failure, history of AMI, ACEI, and uric acid (per 20 units). **B** Baseline risk model vs. + TyG-BMI index in the female patients. Baseline risk model includes age, history of AMI, diabetes mellitus, CTO, uric acid (per 20 units), and number of treated vessels. AUC, area under curve

In elderly participants, we assessed the ROC curves of the baseline risk model consisting of traditional risk factors (including age, heart failure, history of AMI, ACEI, and uric acid (per 20 units)) and the model fit based on the traditional risk factors and the TyG-BMI index (Fig. 3A). There is no significant difference between the baseline risk model (AUC: 0.687) and the model with the TyG-BMI index (AUC: 0.694) (p for comparison = 0.313). We further calculated the more sensitive category-free NRI of 0.221 (p = 0.020) and IDI of 0.006 (p = 0.059) (Table 9). The results showed limited ability to add the TyG-BMI index to the baseline model. In female patients, we assessed the ROC curves of the base model (including age, previous AMI, diabetes mellitus, CTO, uric acid (per 20 units), and the number of treated vessels) and the model consisting of traditional risk factors and the TyG-BMI index (Fig. 3B). No significant improvement was observed in the female patients considering the comparison of AUCs (base model 0.759 vs. base model + TyG-BMI index 0.765, p for comparison = 0.508). The category-free NRI of 0.222 (p = 0.106) and IDI of 0.012 (p = 0.102) indicated the addition of the TyG-BMI index had no significant incremental effect for predicting MACCEs as well (Table 10).

**Table 9:** NRI and IDI for the incremental predictive values of models in elderly patients

	Category-free NRI		IDI	
	Estimate (95% CI)	P value	Estimate (95% CI)	P value
Baseline risk model		Reference		Reference
** + **TyG-BMI Index	0.221(0.035–0.407)	0.020	0.006 (– 0.0003 to 0.013)	0.059

NRI net reclassification index; IDI integrated discrimination improvement

Baseline risk model was adjusted for age, heart failure, previous AMI, ACEI, uric acid (per 20 units)

**Table 10:** NRI and IDI for the incremental predictive values of models in female patients

	Category-free NRI		IDI	
	Estimate (95% CI)	P value	Estimate (95% CI)	P value
Baseline risk model		Reference		Reference
** + **TyG-BMI Index	0.222(– 0.047 to 0.491)	0.106	0.012 (–0.002 to 0.026)	0.102

NRI net reclassification index; IDI integrated discrimination improvement

Baseline risk model was adjusted for age, previous AMI, diabetes mellitus, CTO, uric acid (per 20 units), number of treated vessels

## Discussion

Our research examined the correlation between the TyG-BMI index and MACCEs among CAD patients undergoing PCI with DES. The primary findings were as follows: (1) The TyG-BMI index was substantially linked with MACCEs in elderly patients and female patients; (2) a higher TyG-BMI index was proportionally related to a higher risk of MACCEs in the elderly patients and in the female patients, even when controlling for confounders rel ated to this research; and (3) the inclusion of the TyG-BMI index could not significantly improve the risk prediction for MACCEs either in the elderly patients or in the female patients.

It is well established that insulin resistance (IR) is a prominent risk factor for T2DM, dyslipidemias, obesity, and cardiovascular disease [27]. Previous studies have indicated that IR is associated with the progression of cardiovascular disorders and predicts cardiovascular outcomes [28]. Consequently, the TyG index was developed to assess IR [7, 11]. Moreover, BMI acts as an indicator of obesity and IR. Then, research in the Chinese population revealed that TyG-BMI was well associated with HOMA-IR in non-diabetic patients [22]. The Korean National Health and Nutrition Examination Survey (KNHANES) indicated the superiority of the TyG-BMI index over other parameters for IR assessed by HOMA-IR [6]. Subsequently, the TyG-BMI index was considered a marker of IR. Additionally, the TyG-BMI index was shown to be linked to hypertension and hyperuricemia [29–32]. A cross-sectional survey suggested that the TyG-BMI index and ischemic stroke seemed to be independently associated linearly among the general population, without a threshold or saturation effect [23]. And this implied a potential impact of the TyG-BMI index on MACCEs. Hence, we performed an analysis to obtain an accurate association.

In this study, we adjusted the conventional and possible risk factors initially described in our investigation. Intriguingly, we didn’t discover a remarkable link between the TyG-BMI index and MACCEs among the whole study population. It should be noted that the incidence of MACCEs in the study population was less frequent than that in the excluded population (13.6% vs. 21.2%) (Additional file 1: Table S1). The difference mainly resulted from cardiac death and repeat revascularization, possibly due to the lower proportion of follow-up coronary angiography in the study population (14.3% vs. 36.4%). Besides, the present study demonstrated a lower incidence of MACCEs compared to previous studies. Yang et al. and Zhang et al. reported 375 (34.4%) and 856 (27.0%) major adverse cardiovascular events during follow-up, respectively, in patients with AMI [33, 34]. In patients with both type 2 diabetes and acute coronary syndromes (ACS), 188 (24.2%) MACCEs were observed during follow-up [35]. Patients with stable angina or without diabetes were also included in this study, which may partially explain the lower frequency of MACCEs. Therefore, the result should be considered conservative.

A rising number of studies have focused on cardiovascular risk in the female population, demonstrating that the clinical features and prognosis of CAD patients vary by gender, with female patients likely to have worse clinical outcomes [36, 37]. The TyG index (another indicator of IR) was associated with the incidence of myocardial infarction in the general population after 11 years of follow-up, and the risk was considerably greater in women than in men [38]. Another study revealed gender disparities in the association between several IR evaluation parameters and atherosclerosis [39]. Age is generally accepted to be an independent risk factor for cardiovascular disease, and older patients with CAD have a higher risk of adverse cardiovascular outcomes, including ischemic and bleeding events [40]. The TyG-BMI index has been proven to be related to conventional risk factors, including hypertension and diabetes, and populations with combined hypertension and/or diabetes have a greater incidence of cardiovascular events [3, 4, 29, 41]. Thus, we performed an exploratory subgroup analysis focusing on gender, age, hypertension, and diabetes mellitus. The results demonstrated that higher TyG-BMI indexes were related to an increased risk of MACCEs in the older patients and female patients instead of the overall patients, though the precise mechanism remains unknown. Remarkably, the TyG-BMI index, which consists of three classic cardiovascular disease risk markers, lipid-related, glucose-related, and obesity-related components, is a reliable predictor of IR. Moreover, the TyG-BMI index is a valid indicator of IR, which may partially account for the connection seen in our research. IR can induce glucose metabolic disorders, leading to hyperglycemia and eventually triggering inflammation and oxidative stress [42]. Several studies have revealed that IR may contribute to the inactivation of nitric oxide (NO) and generate the overproduction of reactive oxidative stress (ROS) that damages endothelial function [43]. Furthermore, IR may lead to platelet hyperactivity and increase tissue factor (TF) synthesis, which are associated with thrombosis and inflammation [44]. Moreover, IR triggers inordinate glycosylation, promoting the proliferation of vascular smooth muscle cells and the crosslinking and deposition of collagen, ultimately resulting in diastolic left ventricular stiffness and cardiac fibrosis [28]. Finally, higher TG levels may induce raised plasma free fatty acid (FFA) levels, a major cause of obesity-associated IR and cardiovascular disease [21, 45].

Our findings indicated a relationship between the TyG-BMI index and MACCEs in the elderly population. On the one hand, the physiological functions of elderly people decline, and they become more susceptible to multiple metabolic disorders. This process is often accompanied by IR, which in turn exacerbates metabolic disorders, ultimately causing pathological and physiological changes such as endothelial dysfunction, inflammation, and excessive platelet activation, increasing the risk of cardiovascular disease [40]. On the other hand, compared to younger patients, elderly patients may have multiple risk factors and a worse prognosis. In this research, 137 (17.3%) MACCEs occurred in the elderly patients, which was much higher than the 58 (9%) MACCEs in the non-elderly patients. As a result, the conclusions may not be as definitive as they could be, and further research is needed to analyze the relationship between the TyG-BMI index and MACCEs in non-elderly patients. Meanwhile, we noticed similar effects in the gender subgroup. Further analysis revealed a linear association between TyG-BMI and MACCEs in female patients but not in male patients. Several studies about the relationship between IR and MACCEs have reported gender differences [38, 46]. In this study, the mean age of female patients was higher than that of male patients (63.2 years vs. 58.6 years). This may be due to the protective effects of estrogen on the cardiovascular system and insulin sensitivity [36]. Approximately 90% of the women in this study were in the menopausal transition or postmenopausal period, when the protective effects of estrogen were diminished or disappeared and the risk of abdominal fat gain, dyslipidemia, endothelial dysfunction, and IR was increased [47, 48]. Therefore, a higher variability of the TyG-BMI index was observed in the female patients, which may partly explain the findings in the gender subgroup. However, more studies are needed to verify the association and investigate the exact mechanism. Furthermore, we also investigated the association between the TyG-BMI index and individual cardiovascular events. However, no significant differences were observed between the lower and higher TyG-BMI index groups in the elderly and female patients. According to the original research, 11.4% of the patients with angina during the follow-up accepted conservative therapy with medication, and only 23.8% received follow-up coronary angiography [24]. As a result, the low incidence of the individual events was recorded, which limited the conclusions we could draw from our results. When added to the traditional models, no cumulative effects of the TyG-BMI index were observed according to the AUC, NRI, and IDI. The average age of both elderly and female patients in this study is over sixty, which is a time when multiple traditional cardiovascular risk factors are often combined. This weakens the predictive power of the TyG-BMI index.

As far as we know, this study explored the influence of the TyG-BMI index on the incidence of MACCEs in patients treated with DES for the first time. The current research revealed that the TyG-BMI index and MACCEs had a linear connection in elderly patients and female patients. However, there are several non-negligible limitations. Firstly, no conclusions can be drawn about causality due to the single-center observational design of the study. Secondly, the exclusion of a considerable number of patients due to missing data and the low incidence of cardiovascular events might have underestimated the effect. Therefore, further investigation and validation via large-sample, multicenter studies are needed. Thirdly, we couldn’t compare the TyG-BMI index with other current IR measuring techniques because of database restrictions. Fourthly, triglycerides, glucose, and other parameters were only tested at baseline, and they may have altered over follow-up owing to the participants’ lifestyles and medications. The residual or unmeasured confounding, especially the treatment for metabolic syndrome, dietary habits, and physical activities, were not included. Besides, the time-to-event analysis was not conducted due to the lack of a time variable in the data. These might weaken the results. Finally, the population covered in the study comes from a single site in China, limiting the applicability of the results to other communities. Hence, more research is required to verify these findings.

## Conclusions

In summary, as a marker of IR, the TyG-BMI index was shown to be an independent prognostic factor of MACCEs in the elderly and female patients after DES implantation. A higher TyG-BMI index was proportionally related to an increased incidence of MACCEs in the elderly and female patients. However, the inclusion of the TyG-BMI index did not improve risk prediction over traditional risk factors in the elderly or female patients. Further investigation is needed to demonstrate the findings.

## Supplementary Information

**Additional file 1: Table S1.** Characteristics of the included population and excluded population. Data are shown as mean ± standard deviation (SD) or median (IQR) for continuous variables and proportions (%) for categorical variables. P values in bold are < 0.05. ACEI, angiotensin converting enzyme inhibitor; AVB, atrioventricular block; BMI, body mass index; CCB, calcium channel blocker; CABG, coronary artery bypass graft; COPD, chronic obstructive pulmonary disease; CTO, chronic total occlusions; HDL-C, high density lipoprotein cholesterol; LAD, left anterior descending; LDL-C, low density lipoprotein cholesterol; LM, left main coronary artery; LCX, left circumflex artery; LVEF, left ventricular ejection fraction; NSTE-ACS, non-ST elevation acute coronary syndromes; AMI, acute myocardial infarction; PCI, percutaneous coronary intervention; RCA, right coronary artery; SA, stable angina; STEMI, ST-segment myocardial infarction; TC, total cholesterol; TG, triglyceride. **Table S2.** Selection of covariates and analysis of collinearity in overall patients. Dependent variable: TyG-BMI index. GVIF, generalized variance inflation factor; DF, degree of freedom. (GVIF^(1/(2*Df)) ≥ 2 indicates collinearity). Collinearity analysis showed that BMI, TC, TG, LDL-C, LCX, RCA, length of stents, and the TyG-BMI index had high collinearity. Abbreviations as in Additional file 1: Table S1. **Table S3.** Selection of covariates and analysis of collinearity in elderly patients. Dependent variable: TyG-BMI index. GVIF, generalized variance inflation factor; DF, degree of freedom. (GVIF^(1/(2*Df)) ≥ 2 indicates collinearity). Collinearity analysis showed that BMI, TC, TG, LDL-C, RCA, number of diseased vessels, length of stents, and the TyG-BMI index had high collinearity. Abbreviations as in Additional file 1: Table S1. **Table S4.** Selection of covariates and analysis of collinearity in female patients. Dependent variable: TyG-BMI index. GVIF, generalized variance inflation factor; DF, degree of freedom. (GVIF^(1/(2*Df)) ≥ 2 indicates collinearity). Collinearity analysis showed that BMI, glycemia, TC, TG, LDL-C, LAD, LCX, RCA, number of diseased vessels, length of stents, and the TyG-BMI index had high collinearity. Abbreviations as in Additional file 1: Table S1.

## Data Availability

The datasets generated and/or analyzed during the current study are available in the Dryad repository https://doi.org/10.5061/dryad.13d31 [25].

## Abbreviations

ACEI: Angiotensin converting enzyme inhibitor
AMI: Acute myocardial infarction
AVB: Atrioventricular block
BMI: Body mass index
CCB: Calcium channel blocker
CABG: Coronary artery bypass graft
COPD: Chronic obstructive pulmonary disease
CTO: Chronic total occlusions
DBP: Diastolic blood pressure
HDL-C: High density lipoprotein cholesterol
LAD: Left anterior descending
LDL-C: Low-density lipoprotein cholesterol
LM: Left main coronary artery
LCX: Left circumflex artery
LVEF: Left ventricular ejection fraction
NSTE-ACS: Non-ST elevation acute coronary syndromes
PCI: Percutaneous coronary intervention
RCA: Right coronary artery
SA: Stable angina
SBP: Systolic blood pressure
STEMI: ST-segment myocardial infarction
TC: Total cholesterol
TG: Triglyceride
TyG-BMI index: Triglyceride glucose-body mass index
