# Supplementary material for: Association between triglyceride glucose-body mass index and cardiovascular outcomes in patients undergoing percutaneous coronary intervention: a retrospective study
Source: Cardiovasc Diabetol. 2023 Mar 30;22:75. doi: 10.1186/s12933-023-01794-8 (PMC10064664; doi:10.1186/s12933-023-01794-8)
Supplement: Supplementary file 1 — Additional file 1: Table S1. Characteristics of the included population and excluded population. Data are shown as mean ± standard deviation (SD) or median (IQR) for continuous variables and proportions (%) for categorical variables. P values in bold are < 0.05. ACEI, angiotensin converting enzyme inhibitor; AVB, atrioventricular block; BMI, body mass index; CCB, calcium channel blocker; CABG, coronary artery bypass graft; COPD, chronic obstructive pulmonary disease; CTO, chronic total occlusions; HDL-C, high density lipoprotein cholesterol; LAD, left anterior descending; LDL-C, low density lipoprotein cholesterol; LM, left main coronary artery; LCX, left circumflex artery; LVEF, left ventricular ejection fraction; NSTE-ACS, non-ST elevation acute coronary syndromes; AMI, acute myocardial infarction; PCI, percutaneous coronary intervention; RCA, right coronary artery; SA, stable angina; STEMI, ST-segment myocardial infarction; TC, total cholesterol; TG, triglyceride. Table S2. Selection of covariates and analysis of collinearity in overall patients. Dependent variable: TyG-BMI index. GVIF, generalized variance inflation factor; DF, degree of freedom. (GVIF^(1/(2*Df)) ≥ 2 indicates collinearity). Collinearity analysis showed that BMI, TC, TG, LDL-C, LCX, RCA, length of stents, and the TyG-BMI index had high collinearity. Abbreviations as in Additional file 1: Table S1. Table S3. Selection of covariates and analysis of collinearity in elderly patients. Dependent variable: TyG-BMI index. GVIF, generalized variance inflation factor; DF, degree of freedom. (GVIF^(1/(2*Df)) ≥ 2 indicates collinearity). Collinearity analysis showed that BMI, TC, TG, LDL-C, RCA, number of diseased vessels, length of stents, and the TyG-BMI index had high collinearity. Abbreviations as in Additional file 1: Table S1. Table S4. Selection of covariates and analysis of collinearity in female patients. Dependent variable: TyG-BMI index. GVIF, generalized variance inflation factor; DF, degree of [file 12933_2023_1794_MOESM1_ESM.docx]

**Table S1 Characteristics of the included population and excluded population**

|  | **Included (n = 1438)** | **Excluded (n = 1084)** | | **p value** |
| --- | --- | --- | --- | --- |
| **Demographics** |  |  |  | |
| Gender, female, (%) | 452 (31.4) | 353 (32.6) | 0.546 | |
| Age, years | 60.1 ± 11.1 | 59.8 ± 11.1 | 0.598 | |
| **Medical history** |  |  |  | |
| Heart failure, (%) | 158 (11) | 136 (12.6) | 0.214 | |
| Atrial fibrillation, (%) | 25 (1.7) | 25 (2.3) | 0.311 | |
| Previous AMI, (%) | 129 (9) | 105 (9.7) | 0.54 | |
| COPD, (%) | 11 (0.8) | 11 (1) | 0.504 | |
| Third degree AVB, (%) | 3 (0.2) | 5 (0.5) | 0.301 | |
| Previous stroke, (%) | 63 (4.4) | 72 (6.6) | **0.013** | |
| Previous PCI, (%) | 80 (5.6) | 89 (8.2) | **0.009** | |
| Previous CABG, (%) | 6 (0.4) | 15 (1.4) | **0.008** | |
| Hypertension, (%) | 719 (50) | 525 (48.5) | 0.449 | |
| Diabetes mellitus, (%) | 328 (22.8) | 193 (17.8) | **0.002** | |
| Smoking, (%) | 493 (34.3) | 319 (29.4) | **0.01** | |
| Clinical presentation |  |  | **< 0.001** | |
| 1 | 173 (12) | 239 (22) |  | |
| 2 | 894 (62.2) | 598 (55.2) |  | |
| 3 | 371 (25.8) | 247 (22.8) |  | |
| LVEF, (%) | 61.1 ± 7.3 | 60.6 ± 7.7 | 0.176 | |
| **Laboratory data** |  |  |  | |
| Glycemia, mmol/L (IQR) | 5.2 (4.7, 6.5) | 5.1 (4.5, 6.2) | **< 0.001** | |
| Creatinine, umol/L (IQR) | 69.0 (58.0, 81.2) | 69.0 (58.0, 81.0) | 0.98 | |
| Uric acid, umol/L (IQR) | 297.0 (245.0, 355.0) | 290.0 (244.0, 347.0) | 0.299 | |
| Bilirubin, umol/L (IQR) | 8.6 (5.9, 11.9) | 8.7 (6.4, 12.2) | 0.078 | |
| TG, mmol/L (IQR) | 4.2 (3.5, 4.9) | 4.1 (3.5, 4.8) | **0.047** | |
| TC, mmol/L (IQR) | 1.6 (1.2, 2.3) | 1.5 (1.1, 2.1) | **0.001** | |
| HDL-C, mmol/L (IQR) | 1.0 (0.8, 1.2) | 1.0 (0.8, 1.2) | **0.001** | |
| LDL-C, mmol/L (IQR) | 2.6 (2.1, 3.3) | 2.5 (1.9, 3.1) | **< 0.001** | |
| **Treatment** |  |  |  | |
| Aspirin, (%) | 1416 (98.6) | 1071 (98.8) | 0.672 | |
| Clopidogrel, (%) | 1390 (96.7) | 1030 (95.3) | 0.078 | |
| Beta blocker, (%) | 1002 (69.7) | 716 (66.1) | 0.053 | |
| ACEI, (%) | 838 (58.3) | 516 (47.6) | **< 0.001** | |
| CCB, (%) | 352 (24.5) | 246 (22.7) | 0.297 | |
| Statin, (%) | 1343 (93.4) | 953 (87.9) | **< 0.001** | |
| Radial artery access, (%) | 1406 (97.8) | 1052 (97) | 0.251 | |
| Number of diseased vessels |  |  | **0.02** | |
| 1-vessel disease, (%) | 531 (36.9) | 456 (42.4) |  | |
| 2-vessel disease, (%) | 547 (38) | 379 (35.2) |  | |
| 3-vessel disease, (%) | 360 (25) | 241 (22.4) |  | |
| Location of target lesions |  |  |  | |
| LM, (%) | 46 (3.2) | 39 (3.6) | 0.583 | |
| LAD, (%) | 1203 (83.7) | 881 (81.3) | 0.118 | |
| LCX, (%) | 710 (49.4) | 508 (46.9) | 0.212 | |
| RCA, (%) | 727 (50.6) | 522 (48.2) | 0.232 | |
| **Characteristics of lesions** |  |  |  | |
| Occlusion, (%) | 195 (13.6) | 134 (12.4) | 0.376 | |
| CTO, (%) | 120 (8.3) | 106 (9.8) | 0.212 | |
| Ostial lesion, (%) | 170 (11.8) | 104 (9.6) | 0.075 | |
| Bifurcation lesion, (%) | 249 (17.3) | 196 (18.1) | 0.618 | |
| Restenosis, (%) | 15 (1) | 19 (1.8) | 0.126 | |
| Number of treated vessels, (%) |  |  | 0.84 | |
| 1 | 833 (57.9) | 621 (57.3) |  | |
| 2 | 478 (33.2) | 360 (33.2) |  | |
| 3 | 127 (8.8) | 103 (9.5) |  | |
| Number of stents, (%) |  |  | 0.072 | |
| 1 | 565 (39.3) | 414 (38.2) |  | |
| 2 | 438 (30.5) | 298 (27.5) |  | |
| 3 | 435 (30.3) | 372 (34.3) |  | |
| Length of stents, (mm) | 48.8 ± 31.3 | 51.8 ± 34.2 | **0.022** | |
| Diameter of stents, (mm) | 3.1 ± 1.1 | 3.1 ± 0.4 | 0.116 | |
| Follow-up angiography | 205(14.3) | 395(36.4) | **< 0.001** | |
| **Clinical outcomes** |  |  |  | |
| MACCEs, (%) | 195 (13.6) | 230 (21.2) | **< 0.001** | |
| All cause death, (%) | 90 (6.3) | 95 (8.8) | **0.017** | |
| Cardiac death, (%) | 6 (0.4) | 56 (5.2) | **< 0.001** | |
| AMI, (%) | 58 (4) | 60 (5.5) | 0.077 | |
| Revascularization, (%) | 79 (5.5) | 120 (11.1) | **< 0.001** | |
| Stroke, (%) | 21 (1.5) | 17 (1.6) | 0.826 | |

Data are shown as mean ± standard deviation (SD) or median (IQR) for continuous variables and proportions (%) for categorical variables. P values in bold are < 0.05.

ACEI, angiotensin converting enzyme inhibitor; AVB, atrioventricular block; BMI, body mass index; CCB, calcium channel blocker; CABG, coronary artery bypass graft; COPD, chronic obstructive pulmonary disease; CTO, chronic total occlusions; HDL-C, high density lipoprotein cholesterol; LAD, left anterior descending; LDL-C, low density lipoprotein cholesterol; LM, left main coronary artery; LCX, left circumflex artery; LVEF, left ventricular ejection fraction; NSTE-ACS, non-ST elevation acute coronary syndromes; AMI, acute myocardial infarction; PCI, percutaneous coronary intervention; RCA, right coronary artery; SA, stable angina; STEMI, ST-segment myocardial infarction; TC, total cholesterol; TG, triglyceride.

**Table S2 Selection of covariates and analysis of collinearity in overall patients**

|  | Forward selection | | Backward elimination | | GVIF | DF | GVIF^(1/(2*Df)) |
| --- | --- | --- | --- | --- | --- | --- | --- |
|  | Coefficient | Change percentage | Coefficient | Change percentage |  |  |  |
| Crude/Full | 0.11 | Ref. | 0.01 | Ref. | 1.628 | 1 | 1.276 |
| Gender | 0.11 | 0 | 0.03 | 145.2 | 1.628 | 1 | 1.276 |
| Age | 0.1 | -7.5 | 0.01 | -35.8 | 1.243 | 1 | 1.115 |
| BMI | 0.02 | -84.1 | -0.01 | -98.7 | 23.685 | 1 | **4.867** |
| Heart failure | 0.11 | -5.1 | 0.02 | 12.1 | 1.153 | 1 | 1.074 |
| Atrial fibrillation | 0.11 | 1.4 | 0.01 | -12.5 | 1.098 | 1 | 1.048 |
| Previous AMI | 0.12 | 9.6 | 0.01 | -45.6 | 1.516 | 1 | 1.231 |
| Previous stroke | 0.11 | 0.2 | 0.02 | 74 | 1.126 | 1 | 1.061 |
| Previous PCI | 0.12 | 4.3 | 0.01 | -2.8 | 1.106 | 1 | 1.052 |
| Hypertension | 0.08 | -29 | 0.01 | -54.3 | 1.324 | 1 | 1.151 |
| Diabetes mellitus | 0.07 | -40 | 0.07 | 382.4 | 1.209 | 1 | 1.099 |
| Smoking | 0.13 | 10.7 | 0.01 | -17 | 1.432 | 1 | 1.197 |
| Clinical presentation | 0.11 | -0.9 | 0.02 | 46.9 | 1.846 | 2 | 1.166 |
| LVEF | 0.21 | 81.3 | 0.04 | 185.3 | 1.349 | 1 | 1.162 |
| Glycemia | 0.1 | -12.6 | 0.03 | 102.7 | 1.175 | 1 | 1.084 |
| Creatinine | 0.1 | -12.6 | -1.11 | 3.9 | 1.19 | 1 | 1.091 |
| Uric acid | 0.1 | -15.4 | 0.01 | -10.7 | 1.153 | 1 | 1.074 |
| Bilirubin | 0.09 | -21.2 | 0.03 | 157.7 | 1.08 | 1 | 1.039 |
| TC | 0.1 | -14.7 | 0.03 | 133.3 | 5.843 | 1 | **2.417** |
| TG | 0.11 | 0.8 | -0.38 | -64.4 | 4.233 | 1 | **2.058** |
| HDL.C | 0.12 | 5.2 | 0.02 | 34.1 | 1.292 | 1 | 1.137 |
| LDL.C | 0.1 | -10.7 | 0 | -97.3 | 5.301 | 1 | **2.302** |
| Aspirin | 0.12 | 4.3 | 0.02 | 75.5 | 1 | 1 | 1 |
| Clopidogrel | 0.11 | 0.7 | 0.01 | -25.7 | 1.076 | 1 | 1.037 |
| Beta blocker | 0.11 | -0.9 | 0.01 | -0.8 | 1.116 | 1 | 1.056 |
| ACEI | 0.08 | -32.7 | 0.05 | 243.2 | 1.175 | 1 | 1.084 |
| CCB | 0.11 | 0.7 | 0.02 | 67.5 | 1.293 | 1 | 1.137 |
| Statin | 0.12 | 4.1 | -1.08 | 1.1 | 1.105 | 1 | 1.051 |
| Radial artery access | 0.11 | -0.2 | -1.05 | -1.6 | 1.093 | 1 | 1.046 |
| Number of diseased vessels | 0.1 | -14.2 | 0.01 | 0.2 | 15.251 | 2 | 1.976 |
| LM | 0.12 | 4.3 | 0 | -80.8 | 1.329 | 1 | 1.153 |
| LAD | 0.11 | 1.3 | 0.01 | -4.3 | 2.378 | 1 | 1.542 |
| LCX | 0.11 | -2.2 | 0.01 | -12.3 | 5.76 | 1 | **2.4** |
| RCA | 0.1 | -11.2 | 0.01 | 0.2 | 5.571 | 1 | **2.36** |
| Occlusion | 0.11 | -0.6 | 0.01 | 9.6 | 1.162 | 1 | 1.078 |
| CTO | 0.13 | 13.2 | 0.01 | -0.7 | 1.231 | 1 | 1.11 |
| Ostial lesion | 0.11 | 0.9 | 0.01 | 7.4 | 1.226 | 1 | 1.107 |
| Bifurcation lesion | 0.11 | 0.4 | 0.01 | -6 | 1.241 | 1 | 1.114 |
| Number of treated vessels | 0.11 | 1 | 0.01 | -59 | 3.568 | 2 | 1.374 |
| Number of stents | 0.1 | -11.2 | 0.02 | 21.1 | 5.472 | 2 | 1.529 |
| Length of stents | 0.1 | -13.5 | 0.02 | 58.7 | 4.091 | 1 | **2.023** |
| Diameter of stents | 0.1 | -7.3 | 0.01 | 7.3 | 1.185 | 1 | 1.089 |

Dependent variable: TyG-BMI index.

GVIF, generalized variance inflation factor; DF, degree of freedom. (GVIF^(1/(2*Df)) ≥ 2 indicates collinearity)

Collinearity analysis showed that BMI, TC, TG, LDL-C, LCX, RCA, Length of stents and TyG-BMI index had high collinearity.

Abbreviations as in Table S1

**Table S3 Selection of covariates and analysis of collinearity in elderly patients**

|  | Forward selection | | Backward elimination | | GVIF | DF | GVIF^(1/(2*Df)) |
| --- | --- | --- | --- | --- | --- | --- | --- |
|  | Coefficient | Change percentage | Coefficient | Change percentage |  |  |  |
| Crude/Full | 0.19 | Ref. | -1.31 | Ref. | 62.006 | 1 | 7.874 |
| Gender | 0.11 | 0 | 0.03 | 145.2 | 1.628 | 1 | 1.276 |
| BMI | 0.05 | -74.9 | 0.22 | -116.7 | 46.753 | 1 | **6.838** |
| Age | 0.22 | 16.6 | -1.28 | -2.3 | 1.249 | 1 | 1.118 |
| Heart failure | 0.19 | -0.4 | -1.17 | -10.6 | 1.295 | 1 | 1.138 |
| Atrial fibrillation | 0.19 | 1 | -1.29 | -1.5 | 1.276 | 1 | 1.129 |
| Previous AMI | 0.21 | 10.3 | -1.34 | 1.7 | 1.688 | 1 | 1.299 |
| Previous stroke | 0.19 | 0 | -1.31 | -0.6 | 1.195 | 1 | 1.093 |
| Previous PCI | 0.19 | 1.4 | -1.31 | -0.5 | 1.197 | 1 | 1.094 |
| Hypertension | 0.17 | -10.6 | -1.36 | 3.2 | 1.464 | 1 | 1.21 |
| Diabetes mellitus | 0.16 | -16.2 | -1.01 | -23.1 | 1.576 | 1 | 1.256 |
| Smoking | 0.21 | 12 | -1.26 | -3.7 | 1.597 | 1 | 1.264 |
| Clinical presentation | 0.19 | 0.8 | -1.32 | 0.8 | 2.169 | 2 | 1.214 |
| LVEF | 0.25 | 32.5 | -0.36 | -72.7 | 1.442 | 1 | 1.201 |
| Glycemia | 0.2 | 8 | -0.03 | -97.3 | 3.764 | 1 | 1.94 |
| Creatinine | 0.18 | -2.7 | -1.41 | 7.7 | 1.258 | 1 | 1.122 |
| Uric acid | 0.18 | -3.1 | -1.06 | -19 | 1.302 | 1 | 1.141 |
| Bilirubin | 0.17 | -11.7 | -1.28 | -2.8 | 1.204 | 1 | 1.097 |
| TC | 0.19 | -1.4 | -1.31 | -0.3 | 9.72 | 1 | **3.118** |
| TG | 0.22 | 16.2 | -0.59 | -55.3 | 7.332 | 1 | **2.708** |
| HDL.C | 0.19 | 0.4 | -1.25 | -4.8 | 1.657 | 1 | 1.287 |
| LDL.C | 0.18 | -6.5 | -1.18 | -9.9 | 7.748 | 1 | **2.783** |
| Aspirin | 0.2 | 3.9 | -0.77 | -41.1 | 1 | 1 | 1 |
| Clopidogrel | 0.19 | 0.1 | -1.31 | -0.6 | 1.18 | 1 | 1.086 |
| Beta blocker | 0.19 | -1.8 | -1.23 | -6.2 | 1.194 | 1 | 1.093 |
| ACEI | 0.15 | -20.8 | -1.53 | 16.6 | 1.32 | 1 | 1.149 |
| CCB | 0.2 | 4.3 | -1.32 | 0.1 | 1.348 | 1 | 1.161 |
| Statin | 0.19 | 2.2 | -1.33 | 0.9 | 1.197 | 1 | 1.094 |
| Radial artery access | 0.19 | 0 | -1.22 | -7.4 | 1.24 | 1 | 1.114 |
| Number of diseased vessels | 0.19 | 1.6 | -1.26 | -4.4 | 22.257 | 2 | **2.172** |
| LM | 0.19 | -1.4 | -1.2 | -9 | 1.392 | 1 | 1.18 |
| LAD | 0.2 | 3.7 | -1.39 | 5.8 | 2.994 | 1 | 1.73 |
| LCX | 0.19 | 0.1 | -1.31 | -0.5 | 7.394 | 1 | **2.719** |
| RCA | 0.19 | -1.2 | -1.33 | 0.9 | 7.024 | 1 | 2.65 |
| Occlusion | 0.19 | -0.1 | -1.28 | -2.5 | 1.214 | 1 | 1.102 |
| CTO | 0.21 | 9.9 | -1.36 | 3.8 | 1.388 | 1 | 1.178 |
| Ostial lesion | 0.19 | -1.4 | -1.29 | -1.6 | 1.318 | 1 | 1.148 |
| Bifurcation lesion | 0.19 | -1.2 | -1.32 | 0.8 | 1.318 | 1 | 1.148 |
| Number of treated vessels | 0.2 | 5.4 | -1.29 | -1.5 | 3.78 | 2 | 1.394 |
| Number of stents | 0.17 | -8.4 | -1.41 | 7.5 | 5.498 | 2 | 1.531 |
| Length of stents | 0.18 | -5.6 | -1.28 | -2.7 | 4.238 | 1 | **2.059** |
| Diameter of stents | 0.19 | -1.1 | -1.33 | 1.1 | 1.27 | 1 | 1.127 |

Dependent variable: TyG-BMI index.

GVIF, generalized variance inflation factor; DF, degree of freedom. (GVIF^(1/(2*Df)) ≥ 2 indicates collinearity)

Collinearity analysis showed that BMI, TC, TG, LDL-C, RCA, number of diseased vessels, length of stents and TyG-BMI index had high collinearity.

Abbreviations as in Table S1

**Table S4 Selection of covariates and analysis of collinearity in female patients**

|  | Forward selection | | Backward elimination | | GVIF | DF | GVIF^(1/(2*Df)) |
| --- | --- | --- | --- | --- | --- | --- | --- |
|  | Coefficient | Change percentage | Coefficient | Change percentage |  |  |  |
| Crude/Full | 0.33 | Ref. | 0.14 | Ref. | 71.541 | 1 | 8.458 |
| Age | 0.3 | -9.2 | -0.78 | -674.4 | 1.656 | 1 | 1.287 |
| BMI | 0.68 | 105.7 | 0.1 | -22.6 | 52.835 | 1 | **7.269** |
| Heart failure | 0.31 | -5.6 | 0.21 | 53.6 | 2.038 | 1 | 1.427 |
| Atrial fibrillation | 0.33 | -0.2 | 0.24 | 79.8 | 1 | 1 | 1 |
| Previous AMI | 0.36 | 9.4 | -0.05 | -139.6 | 1.956 | 1 | 1.398 |
| Previous stroke | 0.33 | -0.4 | 0.14 | 6.9 | 1.606 | 1 | 1.267 |
| Previous PCI | 0.33 | 0.6 | 0.12 | -7.6 | 1.614 | 1 | 1.27 |
| Hypertension | 0.34 | 2.5 | 0.03 | -74.7 | 1.98 | 1 | 1.407 |
| Diabetes mellitus | 0.25 | -24.2 | 1.17 | 769 | 2.491 | 1 | 1.578 |
| Smoking | 0.33 | 0.4 | 0.27 | 103.3 | 2.295 | 1 | 1.515 |
| Clinical presentation | 0.35 | 5.9 | -0.11 | -182.9 | 4.751 | 2 | 1.476 |
| LVEF | 0.35 | 5.5 | -0.24 | -277.5 | 1.77 | 1 | 1.33 |
| Glycemia | 0.32 | -2 | 0.83 | 510.8 | 4.351 | 1 | **2.086** |
| Creatinine | 0.33 | -0.4 | 0.07 | -50.3 | 2.02 | 1 | 1.421 |
| Uric acid | 0.35 | 5.3 | 0.73 | 443.3 | 1.935 | 1 | 1.391 |
| Bilirubin | 0.3 | -7.9 | 0.05 | -60.5 | 1.814 | 1 | 1.347 |
| TC | 0.31 | -7 | 0.15 | 8.7 | 13.666 | 1 | **3.697** |
| TG | 0.24 | -25.6 | -0.26 | -293.2 | 8.676 | 1 | **2.946** |
| HDL.C | 0.34 | 2.7 | -0.13 | -196.9 | 2.103 | 1 | 1.45 |
| LDL.C | 0.33 | -0.3 | 0.4 | 195.8 | 12.298 | 1 | **3.507** |
| Aspirin | 0.33 | 0 | 0.11 | -18.2 | 1 | 1 | 1 |
| Clopidogrel | 0.33 | 1.7 | 0.06 | -58.1 | 1.407 | 1 | 1.186 |
| Beta blocker | 0.33 | 0 | 0.17 | 28.7 | 1.351 | 1 | 1.162 |
| ACEI | 0.29 | -11.2 | 0.17 | 26 | 1.831 | 1 | 1.353 |
| CCB | 0.38 | 15.2 | 0.17 | 22.5 | 1.848 | 1 | 1.36 |
| Statin | 0.34 | 2.1 | 0.14 | 1.4 | 1.64 | 1 | 1.281 |
| Radial artery access | 0.34 | 2.8 | -0.12 | -191.4 | 1.618 | 1 | 1.272 |
| Number of diseased vessels | 0.36 | 8.4 | 0.16 | 18.3 | 47.888 | 2 | **2.631** |
| LM | 0.33 | 0.8 | 0.28 | 108 | 1.592 | 1 | 1.262 |
| LAD | 0.34 | 3.8 | 0.12 | -10.2 | 4.668 | 1 | **2.161** |
| LCX | 0.33 | -0.2 | 0.14 | 1.7 | 11.399 | 1 | **3.376** |
| RCA | 0.35 | 5.7 | 0.15 | 11 | 9.702 | 1 | **3.115** |
| Occlusion | 0.32 | -1.8 | 0.25 | 86.3 | 1.54 | 1 | 1.241 |
| CTO | 0.35 | 7.6 | 0.12 | -11.8 | 1.677 | 1 | 1.295 |
| Ostial lesion | 0.33 | 0.4 | 0.16 | 17.5 | 1.73 | 1 | 1.315 |
| Bifurcation lesion | 0.33 | 0.1 | -0.06 | -144.1 | 1.904 | 1 | 1.38 |
| Number of treated vessels | 0.34 | 4.8 | -0.09 | -163.9 | 10.462 | 2 | 1.798 |
| Number of stents | 0.33 | -0.5 | 0.15 | 9.6 | 13.932 | 2 | 1.932 |
| Length of stents | 0.32 | -2.5 | 0.02 | -86.5 | 6.707 | 1 | **2.59** |
| Diameter of stents | 0.32 | -3.6 | 0.13 | -2 | 1.449 | 1 | 1.204 |

Dependent variable: TyG-BMI index.

GVIF, generalized variance inflation factor; DF, degree of freedom. (GVIF^(1/(2*Df)) ≥ 2 indicates collinearity)

Collinearity analysis showed that BMI, glycemia, TC, TG, LDL-C, LAD, LCX, RCA, number of diseased vessels, length of stents and TyG-BMI index had high collinearity.

Abbreviations as in Table S1
